# The Preterm Gut Microbiota: An Inconspicuous Challenge in Nutritional Neonatal Care

**DOI:** 10.3389/fcimb.2019.00085

**Published:** 2019-04-02

**Authors:** Jannie G. E. Henderickx, Romy D. Zwittink, Richard A. van Lingen, Jan Knol, Clara Belzer

**Affiliations:** ^1^Laboratory of Microbiology, Wageningen University and Research, Wageningen, Netherlands; ^2^Department of Medical Microbiology, Leiden University Medical Center, Leiden, Netherlands; ^3^Department of Neonatology, Isala Women and Children's Hospital, Zwolle, Netherlands; ^4^Danone Nutricia Research, Utrecht, Netherlands

**Keywords:** preterm, very low birth weight, gut microbiota, gastrointestinal tract, immune system, growth, development, health

## Abstract

The nutritional requirements of preterm infants are unique and challenging to meet in neonatal care, yet crucial for their growth, development and health. Normally, the gut microbiota has distinct metabolic capacities, making their role in metabolism of dietary components indispensable. In preterm infants, variation in microbiota composition is introduced while facing a unique set of environmental conditions. However, the effect of such variation on the microbiota's metabolic capacity and on the preterm infant's growth and development remains unresolved. In this review, we will provide a holistic overview on the development of the preterm gut microbiota and the unique environmental conditions contributing to this, in addition to maturation of the gastrointestinal tract and immune system in preterm infants. The role of prematurity, as well as the role of human milk, in the developmental processes is emphasized. Current research stresses the early life gut microbiota as cornerstone for simultaneous development of the gastrointestinal tract and immune system. Besides that, literature provides clues that prematurity affects growth and development. As such, this review is concluded with our hypothesis that prematurity of the gut microbiota may be an inconspicuous clinical challenge in achieving optimal feeding besides traditional challenges, such as preterm breast milk composition, high nutritional requirements and immaturity of the gastrointestinal tract and immune system. A better understanding of the metabolic capacity of the gut microbiota and its impact on gut and immune maturation in preterm infants could complement current feeding regimens in future neonatal care and thereby facilitate growth, development and health in preterm infants.

## Background Information

**Figure 1:**
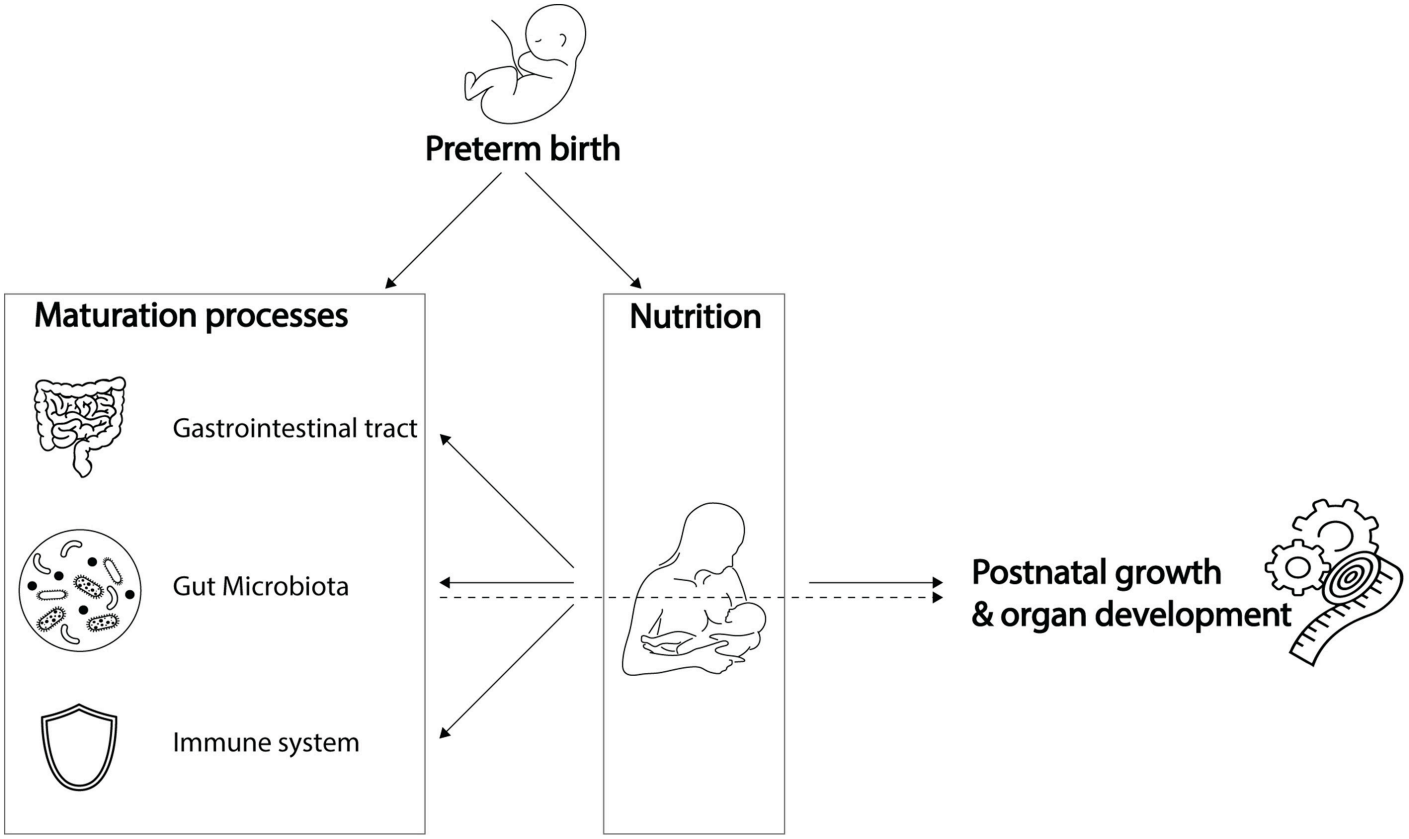
Preterm birth influences breast milk composition and affects maturation processes. Breast milk stimulates maturation of the gastrointestinal tract, gut microbiota and immune system, which, together with its dietary components, promotes post-natal growth and organ development. While preterm birth influences breast milk composition and affects maturation processes, it remains unknown to what extent the preterm gut microbiota is involved in breast milk digestion, and how it contributes to post-natal growth and organ development. Icons were retrieved from The Noun Project. All retrieved icons are licensed as public domain or creative commons (CC BY). Icons were designed by: Cristiano Zoucas (Measuring tape), Design Science (Immune System), Gregor Cresnar (Gears), Jannie Henderickx (baby), Julia Amadeo (Gastrointestinal tract), Julie McMurry (breastfeeding), and Maxim Kulikov (Gut microbiota).

Preterm infants, born before 37 weeks of gestation, are increasingly affected both by prematurity and by complications associated with decreasing gestational age. Complications of prematurity include impaired maturation of the gut microbiota, gastrointestinal tract, and immune system (Figure 1). Yet, simultaneous maturation of the gut microbiota, gastrointestinal tract, and immune system in early life orchestrates further infant growth—that is, weight gain—and organ development. As they are playing a cornerstone role in infant growth and development, impaired maturation of the gut microbiota, gastrointestinal tract, and immune system could have serious health consequences. Preterm infants with extremely low birth weight are susceptible to infections, which in turn is associated with poor neurocognitive functioning (Stoll et al., 2004; Sammallahti et al., 2017). Therefore, preterm infants would benefit from weight gain, implicating growth can be considered as health indicator (Yu et al., 2016; Arboleya et al., 2017). Strict feeding regimens are needed in the neonatal period to stimulate maturation processes, growth, and organ development.

Despite continuous improvements in preterm infant care, optimal feeding for individual infants is challenging. One of the challenges is the differential composition of breast milk associated with preterm delivery (Dallas et al., 2015) (Figure 1). Besides that, the specific nutritional needs of preterm infants are challenging to meet (Neu, 2007a). Another difficulty to achieve optimal feeding regimens is underdevelopment of the gastrointestinal tract that hinders motility and nutrient absorption, factors that might lead to abdominal distension, vomiting and gastric retention (Neu, 2007a). Lastly, underdevelopment of the immune system could trigger exacerbated inflammatory responses to antigens, such as those from undigested food or bacterial compounds, which could contribute to the development of necrotizing enterocolitis (NEC) (Neu, 2007a). As a consequence of these challenges, more than half of the hospitalized preterm infants are being discharged with ongoing severe post-natal growth impairment (Grier et al., 2017).

While meeting nutritional needs is challenging partly due to underdevelopment of the gastrointestinal tract and immune system, there is a gap in knowledge on the involvement of the gut microbiota in meeting nutritional requirements of preterm infants (Figure 1). The gut microbiota has distinct metabolic capacities, making their role in metabolism of dietary components indispensable to the host. In preterm infants, variation in gut microbiota composition is introduced due to a unique set of environmental conditions, including the hospital environment of the Neonatal Intensive Care Unit (NICU) and its associated common clinical practices and feeding regimens. This variation in microbiota composition could interfere directly and indirectly with energy harvest and storage, and thereby with weight gain of the preterm infant (Turnbaugh et al., 2006; Arboleya et al., 2017; Grier et al., 2017).

In this review we hypothesize that variation in gut microbiota composition could have serious consequences on growth and development in preterm infants by differential digestion and absorption of breast milk. We will support this hypothesis by describing the preterm gut microbiota composition and unique environmental conditions contributing to this; and by describing the interaction between breast milk and the gut microbiota, gastrointestinal tract, and immune system.

## A Unique Set of Conditions Shapes the Gut Microbiota of Preterm Infants

**Figure 2:**
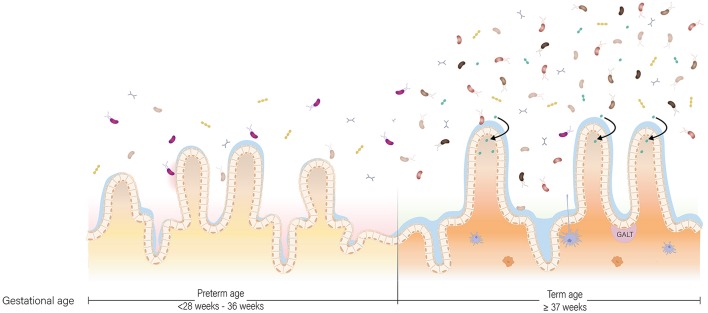
The preterm and term situation of the intestine in early life. In the intestine of infants, maturation of the gut microbiota, gastrointestinal tract and immune system occur at the same time. In the preterm situation, the gut microbiota is low in abundance and in diversity due to the unique set of environmental conditions the infant is exposed to. In the term situation, the gut microbiota is higher in abundance and diversity, and more oriented toward breast milk digestion. The gastrointestinal tract is more mature in term infants compared to preterm infants with regard to enzyme production and activity, nutrient absorption and intestinal motility. Lastly, the preterm situation is characterized by a pro-inflammatory state partly due to a discrepancy in cross-talk between the gut microbiota and immune system, while in the term situation there is oral tolerance.

In early life, the gut microbiota of a term, vaginally-delivered and exclusively breastfed infant is considered the golden standard for a healthy infant microbiota (Arboleya et al., 2015). Generally, the intestine of these infants is colonized with facultative anaerobic bacteria during and shortly after birth due to the presence of low amounts of oxygen in this environment (Penders et al., 2006). These facultative anaerobic bacteria belong to genera *Enterobacter, Enterococcus, Staphylococcus*, and *Streptococcus* (Jacquot et al., 2011). As facultative anaerobic bacteria thrive on residual oxygen in the infant gut, the resulting lowered redox potential allows obligate anaerobic bacteria to proliferate (Penders et al., 2006). *Bifidobacterium, Bacteroides*, and *Clostridium* proliferate and become the predominant genera associated with early life (Thompson-Chagoyán et al., 2007). Further gut microbiota development is driven by host and environmental factors, such as antibiotic treatment, delivery mode, diet and gestational age (Scholtens et al., 2012). Gestational age is among the strongest influencers of gut microbiota development (La Rosa et al., 2014; Korpela et al., 2018). In comparison to term infants, the gut microbiota of preterm infants is characterized by delayed colonization and by limited microbial diversity (Rougé et al., 2010). In addition, levels of commensal, obligate anaerobic bacteria are generally decreased, while levels of potential pathogenic and facultative anaerobic bacteria are increased (Jacquot et al., 2011; Arboleya et al., 2012b; Barrett et al., 2013; Moles et al., 2013) (Figure 2). Comparison of the gut microbiota composition of preterm and term infants showed that *Enterobacter, Enterococcus, Escherichia*, and *Klebsiella* were predominantly present in preterm infants and not so much in term infants (Schwiertz et al., 2003; Arboleya et al., 2012a).

Not only gestational age shapes gut microbiota composition of preterm infants, but an additional unique set of environmental conditions, including the hospital environment, common clinical practices in neonatal care and feeding regimens further contributes to abnormal gut microbiota development (Hartz et al., 2015; Grier et al., 2017).

## The Hospital Environment Converges Differences in Microbiota Composition of Preterm Infants

Environmental conditions are acknowledged for having great influence on bacterial colonization of the intestine (Scholtens et al., 2012). Most preterm infants are exposed to a restricted hospital environment during the first post-natal weeks of life, in which the length of hospital stay is strongly associated with gestational age and bodyweight at birth (Eichenwald et al., 2001; Groer et al., 2014). Not surprisingly, inter-individual differences in microbiota composition of hospitalized very low birth weight (VLBW) infants becomes smaller with increasing stay (Schwiertz et al., 2003; Patel et al., 2016). More specifically, the microbiota of hospitalized VLBW infants converges toward a core microbiota mainly composed of bacterial families *Enterobacteriaceae* (genera *Klebsiella* and *Escherichia* in particular) and *Enterococcaceae* (Patel et al., 2016; Stewart et al., 2016). The NICU-associated core microbiota is very different from healthy term infants, which is commonly composed of *Bifidobacterium, Bacteroides*, and *Clostridium* in early life (Thompson-Chagoyán et al., 2007). In addition to decreased differences in microbiota composition between infants within one care unit, variations in infant microbiota composition and succession between different hospitals have been observed, further supporting the influence of the hospital environment on microbiota composition (Taft et al., 2014). A NICU-specific microbiota composition might be explained by the hospital environment acting as reservoir for microbes, selected by lavish antibiotic use, that subsequently colonize the infant gut (Brooks et al., 2014). Another explanation for a NICU-specific microbiota is transmission of bacteria between patients within one care unit and between patients and caregivers (Almuneef et al., 2001; de Man et al., 2001; Carl et al., 2014). Knowledge on the role of the hospital environment on gut microbiota composition is particularly relevant in preventing colonization with potential pathogenic bacteria, such as *Enterobacter* species that cause outbreaks of nosocomial infections within NICUs (de Man et al., 2001). Among prevalent nosocomial infections are NEC and sepsis, these are both infections in which the gut microbiota has been implicated (Wang et al., 2009; Murgas Torrazza and Neu, 2011; Young et al., 2017).

## Cesarean Delivery Enriches the Gut Microbiota for Skin and Environmental Microbes

The mode of delivery is considered the first and foremost determinant that affects early life microbiota composition (Dominguez-Bello et al., 2010; Collado et al., 2015). The maternal fecal and vaginal microbiota serve as inoculum for the infant's gastrointestinal tract during passage through the birth canal (Houghteling and Walker, 2015b). As such, the gut microbiota of vaginally-delivered infants resembles the maternal fecal and vaginal microbiota, with a dominance of genera *Lactobacillus, Prevotella*, and *Sneathia* (Dominguez-Bello et al., 2010; Collado et al., 2012). In contrast, the microbiota of infants born by cesarean (C)-section is dominated by common skin and environmental microbes, including *Staphylococcus, Propionibacterium*, and *Corynebacterium* (Dominguez-Bello et al., 2010; Collado et al., 2012). Changes in microbial diversity and colonization with specific taxa have been associated with C-section during the first 3 post-natal months (Rutayisire et al., 2016). Although microbiota composition of infants born by natural birth or C-section gradually become similar, differences in abundance and diversity of specific bacterial taxa can remain apparent until 12–24 months of age (Jakobsson et al., 2014; Bäckhed et al., 2015).

More frequently than term infants, preterm infants are born by C-section (Zwittink et al., 2017), thereby contributing significantly to pertubations of their gut microbiota. These perturbations may have health consequences on both short term and long term (Wandro et al., 2018). On short term, pertubations of the gut microbiota as a result of cesarean delivery, may affect developing mucosal and systemic immune functions (Jilling et al., 2006; Dimmitt et al., 2010). Together with limited diversity and pathogen dominance, this leaves preterm infants prone to nosocomial infections, such as NEC and sepsis (Hällström et al., 2004; Cotten et al., 2009; Wang et al., 2009). Long-term consequences, like asthma, allergies, and obesity, are responsible a result of a discrepancy between the simultaneously developing gut microbiota and immune system. Commensal bacteria are responsible for stimulating development of the immune system and for educating which antigens it should respond to or tolerate (Houghteling and Walker, 2015a). Normally, immune responses toward orally administered antigens, including commensal bacteria, are not triggered, a phenomenon known as oral tolerance. Abnormal microbiota development in preterm infants could have long-lasting changes in the way the immune system was programmed, resulting in a “skewed” tolerance that plays a role in diseases, such as asthma, allergies, and obesity (Tamburini et al., 2016). Indeed, these diseases have been related to the changes in microbiota composition upon cesarean delivery (Tamburini et al., 2016).

In attempt to alleviate changes in microbiota composition associated with C-section, pioneer pilot studies transferred vaginal bacteria from mothers to term, cesarean-delivered infants (Dominguez-Bello et al., 2016). This vaginal microbial transfer, or vaginal seeding, partially restored the infant's gut, oral, and skin microbiota to become more similar to the microbiota of vaginally-delivered infants (Dominguez-Bello et al., 2016). Albeit of great potential to beneficially alter the gut microbiota, vaginal seeding has not yet been performed in preterm infants. There is a great need to further assess the ratio between benefit and risk of vaginal seeding in infants (Haahr et al., 2018). At the moment, there is a negative advice for extending this practice, because not enough evidence currently exists about the proposed long-term benefits outweighing the costs and potential risks (Haahr et al., 2018).

## Antibiotic Treatment Perturbs Gut Microbiota Development

Antibiotic treatment is one of the most common practices in NICUs for preventing and treating infections and sepsis (Zwittink et al., 2018). Pre- and perinatal antibiotic treatment of the mother has been associated with abnormal gut microbiota establishment in the preterm infant (Arboleya et al., 2016; Kuperman and Koren, 2016). In addition, broad-spectrum antibiotics, such as amoxicillin, ceftazidime, erythromycin, and vancomycin are often administered from birth onwards (Zwittink et al., 2018). While antibiotics decrease mortality and morbidity rates on the one hand, they disrupt gut microbiota development on the other hand (Gibson et al., 2015). Such disruptions are characterized by: (1) decreased bacterial diversity (Dardas et al., 2014; Greenwood et al., 2014); (2) delayed *Bifidobacterium* colonization (Zwittink et al., 2018); and (3) increased presence of antibiotic resistance genes or abundance of multi-drug resistant members of *Klebsiella, Escherichia, Enterobacter*, and/or *Enterococcus* genera (Dardas et al., 2014; Greenwood et al., 2014; Bäckhed et al., 2015; Moles et al., 2015a; Arboleya et al., 2016; Gibson et al., 2016; Zwittink et al., 2018). Not only administration of antibiotics, but also the duration of the treatment has an effect on the gut microbiota (Dardas et al., 2014; Greenwood et al., 2014; Zwittink et al., 2018). For example, microbial diversity decreases significantly with increasing duration of antibiotic treatment in preterm infants (Dardas et al., 2014; Greenwood et al., 2014). In addition, the time to recover from low *Bifidobacterium* abundance prolongs in preterm infants receiving long antibiotic treatment (≥5 days) compared to preterm infants who received short treatment (≤3 days) (Zwittink et al., 2018). The influence of antibiotics is sustained for at least 2 months after termination of treatment (Tanaka et al., 2009).

The disturbance of the gut microbiota development by antibiotic administration may influence crosstalk with the immune system. As such, sustained alterations in gut microbiota composition could have long-lasting consequences for health. In fact, pre- and post-natal antibiotic use increases the risk of disease later in life, such as asthma and other allergic diseases (Marra et al., 2006; Penders et al., 2011; Chu et al., 2015; Metsälä et al., 2015; Zhao et al., 2015). Also other regularly prescribed medication in neonatal health care, like gastric acid-suppressive medication, has been associated with allergic disease in early childhood, possibly by causing intestinal dysbiosis (Mitre et al., 2018).

## Respiratory Support Shifts the Ratio of Facultative to Obligate Anaerobic Bacteria in the Gut

Respiratory support has recently been shown to drive differences in microbiota development between extremely and very preterm infants (Zwittink et al., 2017). Prolonged duration of respiratory support in preterm infants was associated with predominance of fecal aerobic and facultative anaerobic bacteria (Shaw et al., 2015). The presence of aerobic and facultative anaerobic bacteria suggests that respiratory support in the form of positive airway pressure may introduce oxygen in the otherwise anoxic gastrointestinal tract (Moles et al., 2013; Shaw et al., 2015; Zwittink et al., 2017). As a result of an immature gastrointestinal tract, oxygenation of the gastrointestinal tract could also occur through a permeable intestinal epithelium (Shaw et al., 2015). This oxygenation could impede passage and survival of obligate anaerobic bacteria, allowing aerobic and facultative anaerobic bacteria to thrive (Zwittink et al., 2017).

With a shift in the ratio of facultative to obligate anaerobic bacteria, defense against pathogenic bacteria may be impaired. The most relevant nosocomial infectious agents for preterm infants are among facultative anaerobic bacteria (Arboleya et al., 2012c). Obligate anaerobic bacteria prevent bacterial translocation by strengthening the gut mucosal barrier, adhering to the intestinal mucosa, and impeding pathogen invasion (Duffy, 2000). As such, absence or reduction of obligate anaerobic bacteria in the intestine increases the risk of facultative anaerobic bacteria crossing the intestinal barrier (Duffy, 2000). Another effect that accompanies a shift in the ratio of facultative to obligate anaerobic bacteria, is that metabolism may become aerobic in specific niches of the intestine (Brooks et al., 2015). Overall, this could result in aerobic degradation of human milk or infant formula instead of anaerobic fermentation (Brooks et al., 2015), which presumably affects production of energy, nutrients and bioactive compounds.

## Glycosylated Compounds in Breast Milk Are Affected by Preterm Delivery

Breast milk is the preferred source of nutrition for preterm infants because of its immunological and nutritional benefits. Besides that, mother's own breast milk contains prebiotic and probiotic components and thereby has the ability to shape the infant's microbiota (Cong et al., 2016; Ho and Yen, 2016). In absence of mother's breast milk, preterm infants receive pasteurized donor breast milk as alternative (Parra-Llorca et al., 2018). Recently, also pasteurized donor breast milk has been shown to shape the microbiota by favoring a gut microbiota composition more similar to breastfed infants compared to formula fed infants (Parra-Llorca et al., 2018). Yet, more research is needed to investigate the impact of pasteurized donor breast milk on the preterm infant's gut microbiota composition and its potential biological implications.

In mother's own milk, human milk oligosaccharides (HMOs) are prebiotic components belonging to a group of glycosylated compounds in breast milk called glycans. They comprise a collection of structurally complex sugars that display an array of α-linkages and β-linkages (Dallas et al., 2012a; Pacheco et al., 2015). Particularly *Bifidobacterium* species, but also some *Bacteroides* species, have genes encoding for enzymes required for HMO digestion (Bode, 2012; Garrido et al., 2013). The breast milk of mothers who deliver preterm is much more variable in HMO composition and percentage of fucosylated HMOs compared to mothers delivering at term (De Leoz et al., 2012). Bacteria thriving on selective HMOs will be affected by this higher variation in fucosylated HMOs, which is supported by findings showing that colonization by *Bifidobacterium breve* in the preterm infant's gut was influenced by HMO fucosylation (Underwood et al., 2017). In addition, fucosylated HMOs prevent intestinal bacterial adhesion to epithelial surfaces (De Leoz et al., 2012) and can have an impact on the gut microbiota composition as such (Underwood et al., 2015).

Digestion of HMOs results in production of short-chain fatty acids (SCFAs) that not only serve as energy source for the infant, but also lower luminal pH that subsequently inhibits potential pathogens from colonizing (van Limpt et al., 2004; Martin et al., 2010). Like HMOs, SCFAs are thus involved in managing gut microbiota composition. In preterm infants it has been shown that the total fecal SCFA concentrations increased with gestational or post-natal age, regardless of diet (Favre et al., 2002; Pourcyrous et al., 2014). However, it remains unknown if lower fecal SCFA concentrations in preterm infants is due to lower bacterial production, due to higher uptake by epithelial cells, or both (Favre et al., 2002).

Besides prebiotic components, breast milk has its own (probiotic) microbiota that is mainly composed of bacteria associated with the skin and the intestine (Latuga et al., 2014), like *Bifidobacterium, Staphylococcus, Streptococcus*, and *Pseudomonas* (Martín et al., 2009; McGuire and McGuire, 2017). Many other bacterial genera, such as *Bacteroides, Lactobacillus*, and *Ruminococcus* have been reported in breast milk (Cabrera-Rubio et al., 2012; Latuga et al., 2014; Jiménez et al., 2015; Urbaniak et al., 2016). Methodologic differences in breast milk collection, DNA extraction, amplification and sequencing, and bioinformatics may have contributed to the discrepancy in reported breast milk microbiota composition (McGuire and McGuire, 2017). So far, only few studies have investigated the effect of preterm birth on the milk microbiome, while many more studies have investigated the effect of preterm birth on nutrient composition of breast milk (Montagne et al., 1999). The bacterial composition of preterm vs. term breast milk has been reported to be comparable (Moles et al., 2015b; Urbaniak et al., 2016). The colostrum of mothers who delivered preterm contained *Staphylococcus, Streptococcus*, and *Lactobacillus*, while in more mature milk of the same mothers the genera *Enterococcus* and *Enterobacter* were additionally found (Moles et al., 2015b). Besides changes in composition, bacteria are less abundant in preterm breast milk (Moles et al., 2015b). The enteromammary pathway involves translocation of bacteria by gut monocytes from the gut to mesenteric lymph nodes and mammary glands, and occurs solely in the last weeks before term delivery (Perez et al., 2007; Jeurink et al., 2013). In preterm birth, this pathway is not functional or less active, which results in a reduced absolute abundance of bacteria in breast milk. In addition, mothers who deliver preterm may already receive antibiotics during delivery, which could impact bacterial counts in the mammary glands (Soto et al., 2014). Still, more research is needed to assess the impact of preterm birth on the breast milk-associated microbiota composition and absolute abundance of bacteria.

## Prematurity and Diet Interact With Maturation of the Immune System

While at term birth both the innate and adaptive immune system are not fully functional, they are competent to handle infections and to respond to immunization (Martin et al., 2010). Together with microbiota development, the immune system matures in an age-dependent manner from a Th2-biased immune response toward a balanced Th1/Th2 immune response (Martin et al., 2010). The complete process of immune system maturation and its interaction with the gut microbiota is beyond the scope of this review but is described extensively for the first 1,000 days of life by Wopereis et al. (2014). In short, the gut-associated lymphoid tissue (GALT) is the primary site where the immune system interacts with environmental antigens and commensal bacteria (Wopereis et al., 2014) (Figure 2). These commensal bacteria and their products interact with the host via, for example, Pathogen Recognition Receptors (PRRs) that specifically recognize Microbial Associated Molecular Patterns (MAMPs) or by signaling through G-protein-coupled receptors, such as GPR43 (Wopereis et al., 2014).

Breastfeeding plays a crucial role in immune system development (Agostoni et al., 2009). Besides nutrients, it continuously provides immunological components that promote immune system development (Jackson and Nazar, 2006; Agostoni et al., 2009). Among them are secretory immunoglobulin A (SIgA) (Agostoni et al., 2009; Wopereis et al., 2014); leukocytes—primarily macrophages and neutrophils—that actively engulf microbial pathogens by phagocytosis; and lymphocytes (Jackson and Nazar, 2006). In addition to these components, HMOs interact with the immune system by modulating cytokine production of lymphocytes, subsequently influencing the balance between Th1 and Th2 cells (Bode, 2012). It also reduces selectin-mediated cell-cell interactions and decreases leukocyte rolling on activated endothelial cells (Bode, 2012). This could lead to reduced mucosal leukocyte infiltration and activation (Bode, 2012). Breast milk additionally contains non-specific factors that have antimicrobial and anti-pathogenic effects. These non-specific factors include enzymes and proteins that inhibit growth of many bacterial species by disrupting the proteoglycan layer; and lactoferrin, which limits bacterial growth by removing essential iron (Agostoni et al., 2009). Other components contribute to passive protection in the gastrointestinal tract by preventing adherence of pathogens to the mucosa (Agostoni et al., 2009). A meta-analysis investigating the health benefits of breastfeeding has shown a lower risk of gastrointestinal infection and other diseases in breastfed infants (Agostoni et al., 2009).

Preterm birth has major consequences on immune system development. One consequence of preterm birth is a change in the immunological composition of breast milk. For example, breast milk of mothers who delivered before 32 weeks of gestation contained more SIgA in comparison to mothers who delivered term (Koenig et al., 2005). Higher levels of SIgA in preterm breast milk offer greater protection against infections, implicating compensation for immaturity of the immune system of preterm infants (Koenig et al., 2005). In addition to changes in immunological breast milk composition, immaturity of the immune system is more pronounced in preterm infants compared to term infants. According to Melville and Moss (2013) this immaturity is characterized by: “a smaller pool of monocytes and neutrophils, impaired ability of these cells to kill pathogens, and lower production of cytokines which limits T cell activation and reduces the ability to fight bacteria and detect viruses in cells, compared to term infants”. The immune system of preterm infants also plays a role in NEC, a disease characterized by an exacerbated inflammatory response of the intestines (Martin et al., 2010; Neu and Walker, 2011). In term infants, the response of the innate immune system is biased toward a Th2 phenotype and against Th1-cell-polarizing cytokines (Tamburini et al., 2016). This bias allows for microbial homing and colonization, but also leaves the infant susceptible to opportunistic pathogens shortly after birth (Tamburini et al., 2016). After multiple pathogenic encounters, a time- and age-dependent shift takes place from Th2 toward a balanced Th1/Th2 response (Tamburini et al., 2016). A state of disrupted gut microbiota composition in preterm infants promotes a strong Th1 bias, pushing the immune system to be pro-inflammatory under the influence of IL-12 and IFN-γ secretion, supposedly contributing to NEC (Tamburini et al., 2016) (Figure 2). Another mechanism contributing to gastrointestinal inflammation is disruption of the liver-bile acid-microbiota axis upon alterations in gut microbiota composition (Jia et al., 2017).

## Prematurity and Diet Interact With Maturation of the Gastrointestinal Tract

Structural and functional maturation of the gastrointestinal tract are required for efficient digestion and absorption of nutrients from milk feedings. Development of the gastrointestinal tract during gestation is generally subdivided in processes involved in cytodifferentation, digestion, absorption, and motility (Commare and Tappenden, 2007; Patole, 2013). Anatomically, all parts of the gastrointestinal tract are developed within the first 12 weeks of gestation, while it takes up to 20 weeks for the villi and crypts to develop (Commare and Tappenden, 2007). Many structural and functional properties of the gastrointestinal tract develop within 24 weeks gestation. Digestive enzymes (e.g., lactase, sucrase, maltase, and peptidase) can be detected from 8 weeks gestation, but some enzymes are at that stage far below their full potential concentration and activity (Bourlieu et al., 2014). Lactase activity, important for the degradation of lactose from milk, increases progressively from 24 weeks onwards and reaches maximum activity at 40 weeks gestation (Commare and Tappenden, 2007). Sucking, swallowing, gastric emptying, and intestinal motility develop during the third trimester and effective coordination of these processes is reached at term. Although not yet reaching its full potential, the gastrointestinal tract of infants born at term is ready to receive and process milk feedings. Further maturation of gastrointestinal tract functioning is stimulated by milk feeding itself. This particularly accounts for lactase activity, which rapidly increases from the first milk feeding onwards (Commare and Tappenden, 2007).

In case of preterm birth, the infant particularly suffers from immaturity related to digestion and motility, since these develop during the third trimester (Figure 2). The combination of decreased activity of digestive enzymes, immature motility functions, limited absorptive capacity and increased protein demands in preterm infants, raises a major challenge in meeting their nutritional needs (Neu, 2007a). Preterm infants, particularly those born before 32 weeks gestation, are prone to be intolerant to enteral feeding and therefore nutrients are provided intravenously via parenteral feeding for the first 2–4 weeks. Withholding enteral feeding is not favorable and has been associated with reduced gastrointestinal function and structural integrity. These include a decrease in hormone activity, intestinal mucosa maturation, digestive enzyme activity, nutrient absorption, and motility maturation; and an increase in gut permeability and bacterial translocation (Lucas et al., 1986; Berseth, 1990; Neu, 2007a). To stimulate functional maturation of the gastrointestinal tract of preterm infants, minimal enteral nutrition has been practiced widely in NICUs (Mishra et al., 2008). During minimal enteral nutrition, small volumes (12–24 mL/kg/d) of breast milk or formula are given to the infant, without nutritive intent but aiming to prevent mucosal atrophy and to stimulate gut motility in order to reach full enteral feeding as quick as possible. Breast milk in particular can aid in intestinal maturation, as HMOs in breast milk directly affect intestinal epithelial cells and modulate their gene expression, leading to changes in cell surface glycans and other cell responses (Bode, 2012). Furthermore, the presence of dietary components in the gut lumen is essential for establishing and shaping of the gut microbiota. In turn, bacteria residing in the human gastrointestinal tract play an essential role in metabolism of dietary components, with their metabolic capacity being distinct, but complementary, to the activity of human enzymes (Di Mauro et al., 2013). In addition, the gut microbiota is involved in the degradation of some host-generated compounds, including bile acids and mucus (Rowland et al., 2018). Besides its role in digestion, the gut microbiota plays an essential role in structural development of the gastrointestinal tract. Germ-free mice, among others, have smaller intestinal surface area, decreased epithelial cell turnover, and underdeveloped villi and crypts compared to specific pathogen-free and wild-type mice (Al-Asmakh and Zadjali, 2015). The essential role of gut microbiota in structural development of the gastrointestinal tract has been further supported in a study with preterm infant's gut microbiota, showing that gut microbiota, body weight, and intestinal epithelial development are closely related (Yu et al., 2016). Microbiota transplants from preterm infants with normal weight gain to germ-free mice increased villus height, crypt depth, cell proliferation, and numbers of goblet and Paneth cells when compared to mice inoculated with microbiota from preterm infants with poor weight gain. In addition, tight junctions were enhanced in germ-free mice colonized with microbiota from normal-weight-gain infants (Yu et al., 2016). Although findings in mice cannot be extrapolated to humans directly, it demonstrates that structural development of the gastrointestinal tract is affected by the microbiota. Hence, abnormal microbial colonization of the gut in preterm infants affects the gastrointestinal tract in terms of the intestinal barrier and nutrient absorption.

## The Preterm Gut Microbiota Challenges Nutritional Neonatal Care

As described throughout this review, prematurity and nutrition affect maturation of the gut microbiota, gastrointestinal tract, and immune system. These processes are rather intertwined and consequences of prematurity affect the infant on a systemic level in terms of growth and development.

Preterm infants require adequate feeding and subsequent digestion and absorption of nutrients. However, caretakers have to overcome nutritional challenges in feeding preterm infants to reach optimal growth and development. The first challenge is the high nutritional requirement of preterm infants in particular for protein (Neu, 2007b; Örs, 2013). Even though protein content is higher in preterm breast milk, it still is not sufficient to meet the preterm infant's high nutrient requirements (Örs, 2013; Dallas et al., 2015; Pacheco et al., 2015). Therefore, fortification of preterm breast milk with proteins, minerals, and vitamins is needed to achieve adequate growth and development (Dallas et al., 2012b; Örs, 2013).

Another challenge that caretakers need to overcome in preterm infant feeding is the immature gastrointestinal tract. As a result of ongoing gastrointestinal development, carbohydrate, protein, and lipid digestion does not occur to the full extend in preterm infants (Neu, 2007b) (Figure 2). In case of carbohydrate digestion, most importantly, lactase activity is low in preterm infants; its activity increases from 24 to 40 weeks of gestation (Neu, 2007b). Being built on a basic lactose core, low lactase activity could affect HMO digestion (Bode, 2012; Pacheco et al., 2015). Also mechanisms for protein digestion are underdeveloped in preterm infants. While activity of most milk-derived proteases is not affected by gestational age (Demers-Mathieu et al., 2017), limited gastric acid secretion and low enterokinase activity impedes protein hydrolysis (Neu, 2007b; Demers-Mathieu et al., 2018a). Consequently, preterm infants digest proteins to a lesser extent than term infants (Demers-Mathieu et al., 2018b,c). Lastly, lipid digestion in VLBW infants is affected by lower duodenal concentrations of bile acids that are critical for efficient fat digestion and absorption (Neu, 2007b). Lower duodenal concentrations of bile acids are a result of lower synthesis and ileal reabsorption of bile (Neu, 2007b). After digestion of carbohydrates, proteins, and lipids, subsequent nutrient absorption could additionally be lower. The intestine and thus the absorptive surface is still elongating in the third trimester (Commare and Tappenden, 2007). In addition, hampered motility could lead to retention of undigested content in the intestinal lumen for a considerable longer time period, which may initiate an inflammation cascade (Commare and Tappenden, 2007).

Practical hurdles with regard to nutrient requirements and gastrointestinal prematurity are relatively conspicuous. However, we hypothesize that prematurity of the gut microbiota may be an additional inconspicuous challenge in preterm nutritional care (Figure 2). In a healthy state, the gut microbiota contributes to growth and development in two ways. First, the gut microbiota has a distinct, yet complementary, metabolic capacity to human gastrointestinal enzymes. As a result of bacterial digestion, otherwise unavailable energy and nutrients are provided to the host (Krajmalnik-Brown et al., 2012). Second, the gut microbiota is involved in host body weight management (Ley et al., 2005; Turnbaugh et al., 2006; Jumpertz et al., 2011; Blanton et al., 2016). The gut microbiota manages body weight by being involved in production of metabolites and in the harvest, storage, and expenditure of energy from food components by affecting the intrinsic metabolic machinery of host cells (Hooper et al., 2002; Krajmalnik-Brown et al., 2012). The most convincing involvement of gut microbiota in body weight management is the induction of an impaired growth phenotype upon microbiota transplant from undernourished children to germ-free mice (Blanton et al., 2016). While germ-free mice receiving microbiota from undernourished children showed growth impairment, their littermates receiving microbiota from healthy children showed a healthy phenotype (Blanton et al., 2016). Moreover, the impaired growth phenotype could subsequently be ameliorated by introducing two invasive bacterial species, *Ruminococcus gnavus* and *Clostridium synbiosum* (Blanton et al., 2016).

While several studies suggest the involvement of the gut microbiota in body weight and growth management in adults and children (Cardinelli et al., 2015), little is known about this role in preterm infants. Literature on this topic is scarce and thereby represents a major gap in this field of research. Given that preterm birth impedes “normal” gut microbiota development, the role of the preterm gut microbiota in altered digestion of milk feedings and in gut maturation—and thereby affecting post-natal growth and development—becomes increasingly likely. Even though research is scarce and mechanisms remain unknown, some studies in preterm infants suggest an association between the gut microbiota, growth, and development in early life (Arboleya et al., 2017). Grier et al. (2017) identified microbiota phases in preterm infants that were each characterized by distinct metabolic functions. Significant associations were found between nutrition, microbiota phase and preterm infant growth (Grier et al., 2017). Also Arboleya et al. (2017) associated specific bacterial families and genera with weight gain. Especially *Enterobacteriaceae* and *Streptococcus* levels at 2 days of age and *Bacteroides*-group levels at 10 days of age were associated with weight gain at 1 month of age (Arboleya et al., 2017). In addition to that, some bacterial genera—including *Staphylococcus* and *Enterococcus*—were negatively associated with weight gain, while *Weissella* was positively associated with weight gain in preterm infants (Arboleya et al., 2017). These genera, or specific species or strains within these genera, may affect infant food digestion capacity and subsequent energy harvest (Turnbaugh et al., 2006; Jumpertz et al., 2011; Krajmalnik-Brown et al., 2012). Possible mechanisms of these taxa could be differential abundance of genes involved in metabolism of carbohydrates, proteins, and/or lipids (Grier et al., 2017). In fact, differences have been reported in microbial proteins involved in metabolic activity between preterm infants of varying gestational and post-natal age (Young et al., 2015; Zwittink et al., 2017). Most likely, microbial effects on infant growth are strain-specific, each having distinct genes encoding for proteins involved in metabolism (Brooks et al., 2015; Hays et al., 2016). Besides specific taxa, also microbial diversity appears to play a role in achieving digestive tolerance and weight gain (Jacquot et al., 2011).

Based on these clues in current research, it becomes increasingly likely that prematurity of the gut microbiota may be an additional clinical challenge in achieving optimal feeding. The preterm gut microbiota may have a differential metabolic capacity compared to term infants due to variation in the abundance of genes that are involved in metabolism of carbohydrates, proteins, and/or lipids. By having a differential food digestion capacity and energy harvest, the preterm gut microbiota could be involved in preterm infant weight gain and development as such. We expect that the variation in gut microbiota of preterm infants will be mainly emphasized in digestion of glycosylated carbohydrates (HMOs) and proteins (glycoproteins) from breast milk, since gut bacteria have genes encoding for enzymes that digest these components (Garrido et al., 2013). However, we should not exclude the possibility of changes in the type of bioactive compounds, or in the activity of these compounds, considering that breast milk contains many bioactive compounds and the gut microbiota is involved in their production (Collado et al., 2015). Changes in bioactivity of degraded compounds could subsequently influence the antimicrobial properties or cross-talk with the intestinal epithelium and immune system that manage inflammatory responses. However, to date, it remains undiscovered to what extent HMO and glycoprotein digestion takes place in the preterm intestine, and how the intact or digested compounds contribute to the nutritional value and the health benefits for preterm infants.

## Concluding Remarks

The preterm infant is predisposed to health complications, both on short and long term, due to underdevelopment of the the gut microbiota, gastrointestinal tract, and immune system. Specifically, the gut microbiota of preterm infants is shaped by a unique set of environmental conditions, which we hypothesized as inconspicuous clinical challenge in nutritional neonatal care. Current research provides clues that prematurity affects infant growth and development. Exploration of the metabolic capacity of the preterm gut microbiota, with HMO-degrading *Bifidobacterium* spp. and *Bacteroides* spp. in particular, would contribute to a better understanding of production of energy and metabolites that become available to the preterm infant and impact gut maturation and overall host metabolism. This knowledge could complement current nutritional neonatal care and benefit infant growth, development, and health in the future. As such, the preterm infant gut microbiota remains a research priority, in which a reference for a healthy, preterm microbiota composition and its interactions with the gastrointestinal tract and immune system need to be incorporated to thoroughly understand mechanisms by which the gut microbiota is involved in preterm infant growth, development, and health.

## Author Contributions

JH, JK, and CB defined the topic of the review. JH and RZ wrote the manuscript. CB guided the writing of this manuscript. RZ, RL, JK, and CB provided their input and critically reviewed the manuscript. All authors read and approved the final manuscript.

### Conflict of Interest Statement

JK is employee of Danone Nutricia Research, The Netherlands. JH, JK, and CB are financially supported by Danone Nutricia Research. The remaining authors declare that the research was conducted in the absence of any commercial or financial relationships that could be construed as a potential conflict of interest.

## Abbreviations

C-section: cesarean section
GALT: gut-associated lymphoid tissue
HMO: human milk oligosaccharide
MAMP: microbial associated molecular pattern
NEC: necrotizing enterocolitis
NICU: neonatal intensive care unit
PRR: pathogen recognition receptors
SCFA: short-chain fatty acid
SIgA: secretory immunoglobulin A
VLBW: very low birth weight.
